# THRIVE Conceptual Framework and Study Protocol: A Community-Partnered Longitudinal Multi-Cohort Study to Promote Child and Youth Thriving, Health Equity, and Community Strength

**DOI:** 10.3389/fped.2021.797526

**Published:** 2022-02-04

**Authors:** Anna K. Ettinger, Doug Landsittel, Kaleab Z. Abebe, Jamil Bey, Val Chavis, Judith D. Navratil, Felicia Savage Friedman, Terence S. Dermody, Elizabeth Miller, the Pittsburgh Study Committee Co-Leads

**Affiliations:** ^1^Department of Psychology, Center for Children and Families, University of Pittsburgh, Pittsburgh, PA, United States; ^2^Department of Epidemiology and Biostatistics, School of Public Health, Indiana University, Bloomington, IN, United States; ^3^Division of General Internal Medicine, Department of Medicine, University of Pittsburgh School of Medicine, Pittsburgh, PA, United States; ^4^UrbanKind Institute, Pittsburgh, PA, United States; ^5^Division of Adolescent and Young Adult Medicine, Department of Pediatrics, University of Pittsburgh School of Medicine, Pittsburgh, PA, United States; ^6^YogaRoots on Location, LLC, Boca Raton, FL, United States; ^7^Departments of Pediatrics and Microbiology and Molecular Genetics, University of Pittsburgh School of Medicine, Pittsburgh, PA, United States

**Keywords:** child thriving, community partnered participatory research (CPPR), child health equity, longitudinal study, youth well-being

## Abstract

**Background:**

Given the profound inequities in maternal and child health along racial, ethnic, and socioeconomic lines, strength-based, community-partnered research is required to foster thriving children, families, and communities, where thriving is defined as optimal development across physical, mental, cognitive, and social domains. The Pittsburgh Study (TPS) is a community-partnered, multi-cohort study designed to understand and promote child and youth thriving, build health equity, and strengthen communities by integrating community partners in study design, implementation, and dissemination. TPS launched the Tracking Health, Relationships, Identity, EnVironment, and Equity (THRIVE) Study to evaluate children's developmental stages and contexts from birth through completion of high school and to inform a child health data hub accessible to advocates, community members, educators, health professionals, and policymakers.

**Methods and Analysis:**

TPS is rooted in community-partnered participatory research (CPPR), health equity, antiracism, and developmental science. Using our community-informed conceptual framework of child thriving, the THRIVE Study will assess cross-cutting measures of place, environment, health service use, and other social determinants of health to provide longitudinal associations with developmentally appropriate child and youth thriving outcomes across participants in six cohorts spanning from pregnancy through adolescence (child ages 0-18 years). Data from electronic health records, school records, and health and human services use are integrated to assess biological and social influences of thriving. We will examine changes over time using paired *t*-tests and adjusted linear regression models for continuous thriving scores and McNemar tests and adjusted logistic regression models for categorical outcomes (thriving/not thriving). Data analyses will include mixed models with a random intercept (in combination with the previously-specified types of regression models) to account for within-subject correlation.

**Discussion:**

By enhancing assessment of child and youth well-being, TPS will fill critical gaps in our understanding of the development of child and youth thriving over time and test strategies to support thriving in diverse communities and populations. Through CPPR and co-design, the study aims to improve child health inequities across multiple socioecological levels and developmental domains.

## Background

Racial inequities in maternal and child health and academic outcomes persist across all developmental stages, from pregnancy through adolescence and adulthood. In Pittsburgh, Pennsylvania, for example, infant mortality rates are over four times higher for Black infants and 40% of all Black children live below the Federal poverty level compared to 8% of White^1^ children (1). These inequities, mediated by racism, poverty, and place-based social and structural influences on health, are the biggest threats to childhood health and require innovative solutions for building child and family strengths and assets to provide the support and resources that all children need to thrive. Focusing on child and youth “thriving” or flourishing, rather than risks and adversities, offers a promising strategy to address inequities and foster positive trajectories of child development over time. While emerging research is beginning to explore positive child health (2) and flourishing (3), longitudinal research to examine thriving over time is limited, and additional studies are needed to determine which early thriving indicators may predict future success.

Additionally, beyond elucidating predictors and correlates of thriving, research on interventions that optimally support early thriving across diverse communities is also limited. Providers and health systems increasingly acknowledge the importance of social influences of health by assessing for social risk factors, such as poverty, housing, and food insecurity (4). Yet assessment tools rarely consider or address social and community strengths, such as community resources, supportive relationships, and existing capacity. Centering social and community strengths and assets has potential to enhance child thriving through strength-based, family-centered interventions and through structural, community-level interventions (5).

To promote child/youth thriving, build health equity, and support community strengths in Allegheny County, we formed a broad coalition and established collaborations across academic, health system, local government, and community organizations to develop The Pittsburgh Study (TPS), a community-partnered, longitudinal study. TPS includes multiple cohort intervention studies spanning pregnancy through adolescence and a longitudinal, cross-cutting study, Tracking Health, Relationships, Identity, EnVironment, and Equity (THRIVE). We developed a community-informed, multi-dimensional conceptual framework to guide the study. With racial justice, equity, and inclusion as core principles, TPS involves community members as partners throughout all aspects of the study, including leadership, planning, study design, data collection and analysis, and dissemination of findings. This paper describes our definition and conceptual framework for thriving and the protocol for the TPS THRIVE Study.

### Study Overview

The vision of TPS is that “*Every child in our region is healthy, thriving, and achieving their academic goals.”* Using a strengths-based approach (6) focused on existing resources rather than a traditional deficit model, TPS aims to promote child and adolescent thriving through cross-sector collaborations between health care, public health, social services, education, and other community organizations. TPS THRIVE will follow children in Allegheny County, PA from birth through high school—tracking participants' medical, educational, environmental, and demographic information to define the most important biological and social influences on child and adolescent thriving. Given the community-partnered study structure and focus on developing longitudinal, multidimensional thriving measures from pregnancy through adolescence, TPS is poised to fill critical gaps in our knowledge of what it takes for children and youth to thrive.

### Study Conceptual Framework

TPS THRIVE is guided by a community-informed conceptualization of child and youth thriving based on a concept mapping study with 91 community members and health professionals in multiple neighborhoods (7). Concept mapping is a rigorous mixed-method, community-based research approach that provides a structured process for gaining participatory input on a question of interest and results in a visual display of how participants view a given topic (8, 9). We conducted additional interpretation and validation focus groups with over 150 stakeholders including community leaders, youth, and families to update the conceptual framework.

**Figure 1:**
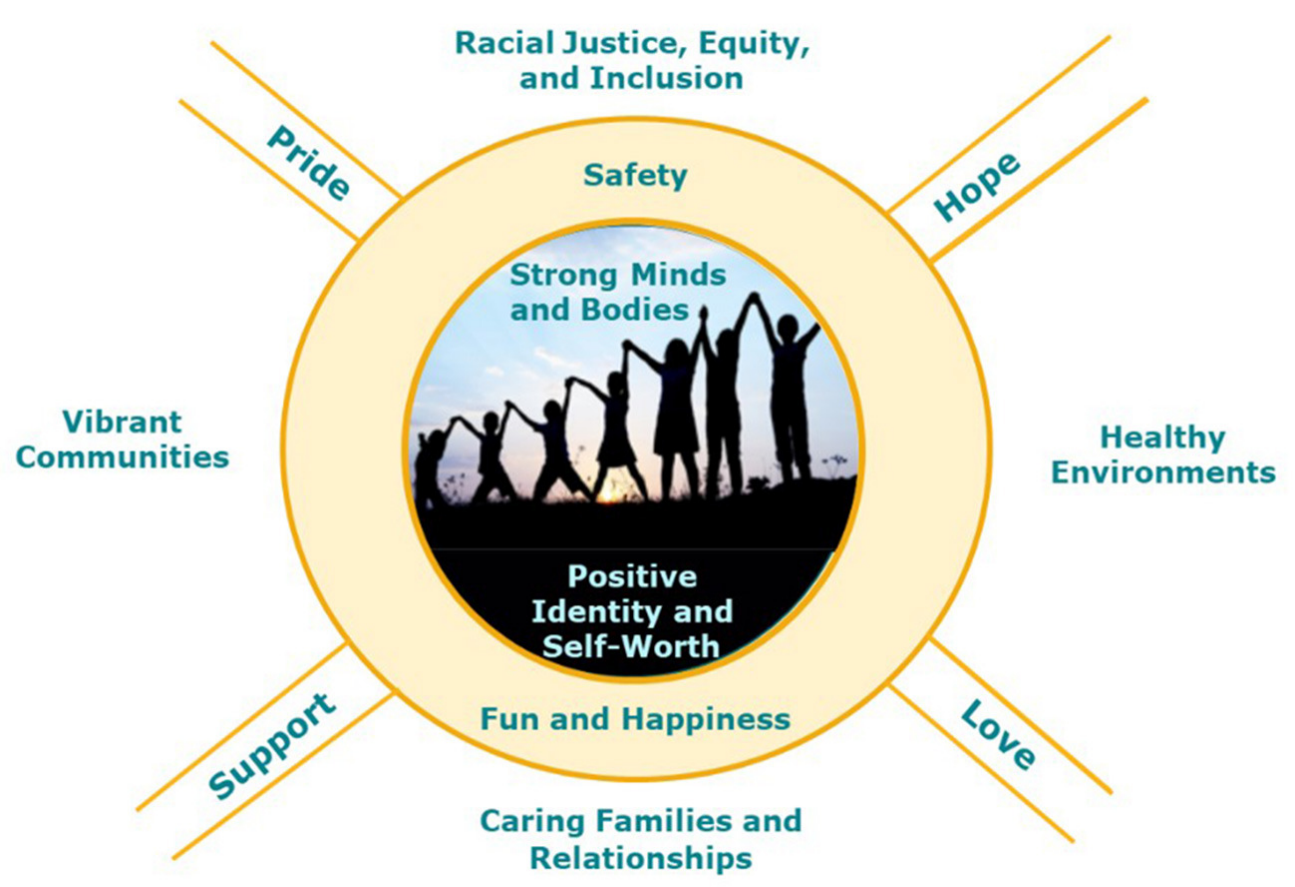
Community-informed conceptual framework of child and youth thriving. This conceptual framework includes eight domains of child and youth thriving across individual, relationship, and contextual levels. Individual-level child development is depicted in the center of the circle, with Strong Minds and Bodies and Positive Identity and Self-worth and the picture of children growing over time. Children/youth are encircled by relationship and contextual factors: Vibrant Communities, Healthy Environments, and Caring Families and Relationships provide safety and opportunities for fun and happiness. Racial Justice, Equity, and Inclusion are foundational practices that support optimal child development. Pride, hope, support, and love were items ranked as most important to child/youth thriving by community and research participants and form rays emanating from thriving children and youth.

Concurrently, we conducted a scoping review of over 12,000 current definitions, frameworks, and assessments of child and youth thriving, including ecological, developmental systems, and equity frameworks. Based on our concept mapping work, our current framework of child thriving identified eight key domains, including child, family, community, and larger environmental influences on child thriving (Figure 1). These domains are described in more detail in Table 1.

**Table 1:** Description of community-informed child thriving domains.

**Domain**	**Description**
Strong minds and bodies	Characterized by positive mental health, cognitive development, physical health, and health behaviors; resources to become a healthy, self-sufficient adult; being thoughtful and positive decision-makers.
Positive identity and self-worth	Positive self-concept, self-efficacy, and social well-being, including children and youth having pride in themselves, hope for the future, and a strong sense of self and self-worth, and developing meaning and purpose.
Fun and happiness	Opportunities for fun, feeling happy, and having positive attitudes about and engagement with the community, as well as access to child-focused community advocates who speak up for children.
Caring families and relationships	Having caring, stable, and positive relationships with parents/caregivers, other family members, teachers, mentors, peers, friends, and partners; positive role models and mentors in addition to parents or caregivers.
Safety	Comprised of safe spaces (schools and neighborhoods), secure relationships (not fearing bullying, violence, or abandonment), and protected development (free to be children).
Vibrant communities	Community and local resources such as community programs; family services; religious institutions; engaging, quality schools and educational programs; and accessible and affordable transportation.
Healthy environments	Encompasses both physical and social environments, including clean air and water; adequate medical, mental health, and social services; economic opportunities; and access to fresh, healthy foods and having food secure households.
Racial justice, equity, and inclusion	Ensuring the systematic fair treatment of children and youth across settings to provide equitable opportunities and outcomes, particularly addressing the experiences of Black children. Children and youth feeling comfortable, accepted, and included in all spaces they enter, regardless of race/ethnicity, gender, religion, health status, and appearance.

We have worked with partners to develop definitions to guide implementation of our study. We defined thriving based on community partner feedback: Child and youth thriving is positive physical, mental, cognitive, and social well-being. Strong minds and bodies, positive identity, feelings of self-worth, and hope for the future characterize thriving. Interactions with caring families and relationships, vibrant communities, and healthy environments provide love, support, safety, and fun and happiness. Racial justice, equity, and inclusion recognize that thriving is an inherent human right of every child. Thriving children are equipped with the resources to accomplish their goals and become successful adults.

Aligned with the National Institute of Minority Health and Health Disparities (NIMHD) Research Framework (2017), the THRIVE study intersects multiple domains of influence (from biological and behavioral to sociocultural and health system) and across multiple levels of health outcomes including individual, family, and community (10). However, our study moves beyond assessing and identifying disparities to engaging our community partners in identifying solutions to address the disparities and inequities in the region, as described in our study objectives and methods.

### Study Objectives

The overall goal of TPS THRIVE is to address critical gaps in our understanding of early childhood thriving across family, school, and community contexts. The study's central hypothesis is that measuring and supporting positive health and thriving, in addition to assessing risk factors and adverse experiences, is essential for promotion of optimal child health and reduction of inequities across diverse families and communities.

#### Primary Research Objectives

The overarching study objectives are:

1. To determine the key biological, psychological, social, community/neighborhood, environmental factors that influence child and youth thriving outcomes across childhood.
2. To identify the effect of policies and place-based factors on childhood thriving through community-partnered and equity-focused policy analysis and outreach.

#### Community-Action Objectives

3. To mobilize community assets to establish environments and communities that promote nurturing, accessible, equitable early childhood experiences across Allegheny County neighborhoods, schools, and service delivery settings.
4. To promote health systems that ensure equitable, easy, and transparent access to trusted, family-centered, culturally-sensitive, and high-quality services that support childhood thriving within TPS.
5. To make community information relevant to child health aligned, available, and accessible through a child health data hub for advocates, community members, educators, health professionals, and policymakers.

### Community Partnerships and Leadership

Multiple institutions have actively aligned around children's health through the Pittsburgh Study, offering an unprecedented opportunity to test innovative interventions to improve child and youth thriving in our community. TPS is jointly directed by the University of Pittsburgh and UPMC Children's Hospital of Pittsburgh along with community partners including the UrbanKind Institute, Urban League of Greater Pittsburgh, Yoga Roots On Location, Inc., the Allegheny County Health Department, Allegheny County Department of Human Services, school systems, family support centers, community non-profits, and local foundations. Partners include the Mayor's Office, advocacy organizations such as Allies for Children, and local and state government agencies that provide opportunities for translating findings into actionable results that can inform local polices and programming to address inequities in child health. These partnerships support the co-design and co-development of research goals and shared decision-making with university researchers.

#### Study Leadership and Core Team

The study is co-directed by an academic Primary Investigator and a community leader. The Core Team provides administrative coordination and support. Core Team members include an Executive Director, Study Coordinator, Senior Research Scientists, and a Community Outreach and Engagement Specialist.

#### Cohort and Cross-Cutting Scientific Committees

TPS is guided by 10 scientific committees (four cross-cutting committees and six cohort committees), comprised of at least 50% community members representing diverse populations and community organizations. The six cohort committees lead individual studies in collaboration with both the cross-cutting committees and study leadership. TPS has established contracts with community members to enable payment for their time, effort, and study contributions. Each of these groups is co-led by academic and community members. The cross-cutting groups focus on policy and neighborhood contexts (Policy and Place), biological and environmental influences on health (Healthy Environments, Strong Bodies), alignment and accessibility of data relevant to child health (Data Accessibility), and health services for children and youth (Health Services Delivery). Additionally, the study is supported by several study infrastructure groups: Equity, Ethics, and Community Accountability (reviews study procedures and results for any adverse events or potential harm to participants and communities); Data Statistical Core (provides statistical consulting and analysis); and Communication (provides outreach, communication, and engagement with the study through multiple dissemination methods).

#### Internal and External Advisory Committees

Given the scope and complexity of TPS, we developed a study infrastructure and transdisciplinary leadership team to provide the breadth and depth of expertise required across relevant parent and child content and research methods. An Internal Advisory Committee comprised of academic and community leaders focused on child health in Allegheny County meet quarterly to provide guidance on study design, progress, and equity of policies and practice. An External Advisory Committee comprised of leading national experts in child and adolescent health, health disparities, and longitudinal intervention research external to Allegheny County provides annual input on the study.

## Methods and Analysis

TPS is rooted in community-partnered participatory research (CPPR) approaches and an antiracist framework (11) to engage with communities, neighborhoods, and families. We recognize community members as equal partners, integrate their expertise into the study, and maintain a commitment to using results to benefit the community. We have incorporated the principles of community engagement (12), community-based participatory research (13, 14), health equity (15–17), and cultural humility (18) to develop the TPS Shared Principles together with our community partners:

1. Connect with communities with honesty, empathy, and transparency.
2. Prioritize community input and recognize that neighborhoods matter.
3. Continue to build trust and show that we care and are fair and consistent.
4. Develop research *with* people not *on* people.
5. Maintain open, inclusive communication—share everything to a fault, including data.
6. Keep learning, listening, and expanding the table.
7. Build collaborations, break down silos.
8. Have patience for the long-term measurable, sustainable impact.
9. Approach decisions with intentional action for impact.
10. Leave your ego at the door.

Recognizing racism as a key driver of child health and academic inequities and outcomes (19, 20), our study centers child health equity, defined by our partners as occurring when: “*All children live in healthy communities, neighborhoods, and environments and receive the support they specifically need to achieve their highest level of well-being.”* To achieve this goal, we have applied an antiracism approach to this research, defined by our community as “*Identifying and disrupting racist practices and promoting actions and ways of being that actively celebrate and support the humanity of all people.”* To intentionally build health equity, we will apply Hogan's (11) Health Equity Framework for research with Black populations to address the history of oppression and racism in the US using the five R4P components: (1) Remove—initiate efforts to identify and undo institutional racism; (2) Repair—address exposures from the past; (3) Remediate—protect children from current risks; (4) Restructure—address structural factors; and (5) Provide—implement actions, programs, and policies to diminish disadvantage. We will accomplish this work through regular antiracist training of study personnel and committees; study oversight by the Equity, Ethics, and Community Accountability Committee that reviews study procedures and activities using a racial equity lens; assessing racism, racist practices, and their implications for child health through study measures; and implementing strategies to challenge racism in our cohort study interventions. Each of our six cohorts is focused on promoting racial equity in child outcomes across the developmental span including maternal and infant birth outcomes, school readiness, reading and math scores, school disciplinary procedures, violence exposure, and academic achievement and high school graduation.

### Study Design and Setting

This study will recruit families living in Allegheny County in western Pennsylvania (PA) which includes the city of Pittsburgh and its surrounding communities. With a population of over 1.2 million residents, most residents are White, with approximately 13% identifying as Black, 4% as Asian, 2% as multiracial or other racial groups, and 2% identifying as Hispanic (1). Although the county's overall poverty rate of 7% of families living below the Federal Poverty Level (FPL) is on par with the state average, 24% of Black families live below the FPL compared with 5% of White families (1). Furthermore, 45% of Black children, and 23% of Hispanic or Latino children relative to 8% of White children under the age of 6 years live below the FPL (1). Income, homeownership, and educational attainment are lower among Black residents relative to White residents in Allegheny County, and Black and Hispanic unemployment are significantly higher than White and Asian rates in the Pittsburgh area (21). Black families are the most segregated group in the Pittsburgh area based on residence and school demographics (21). Over 16% of children in Allegheny County are food insecure (22), ranking among the worst 25% of counties in PA and the US (1). The Child Opportunity Index (23), which quantifies and maps 29 neighborhood conditions related to education, health and environment, ranges from very low to very high opportunities in Pittsburgh depending on the neighborhood, highlighting existing inequalities in child opportunity in areas of higher concentrations of Black and low-income children.

These inequities in important social influences on health drive many of the health and academic disparities in child health observed in Allegheny County. For example, infant mortality rates vary by race from 14.1 deaths per 1,000 live births for Black infants compared to 3.3 deaths per 1,000 live births for White infants (24). Two times as many Black infants were born with low birth weight in 2018 relative to White infants (13.9 vs. 6.2%), and asthma hospitalization rates were over four times higher among Black children than White children in 2018 (25). Complete childhood immunization rates were lower among Black 18-35 month old toddlers compared with White toddlers (55 vs. 68%) (26). According to the 2019 Inequality Across Gender and Race report about the city of Pittsburgh (27), Pittsburgh's high schools are in the bottom 20% for students taking ACT/SATs, and White residents are three times more likely to have a college degree than Black residents. While high school graduation rates are high, 16% of Black men in Pittsburgh do not have a high school diploma or GED, 2.5 times higher than White residents (27).

**Figure 2:**
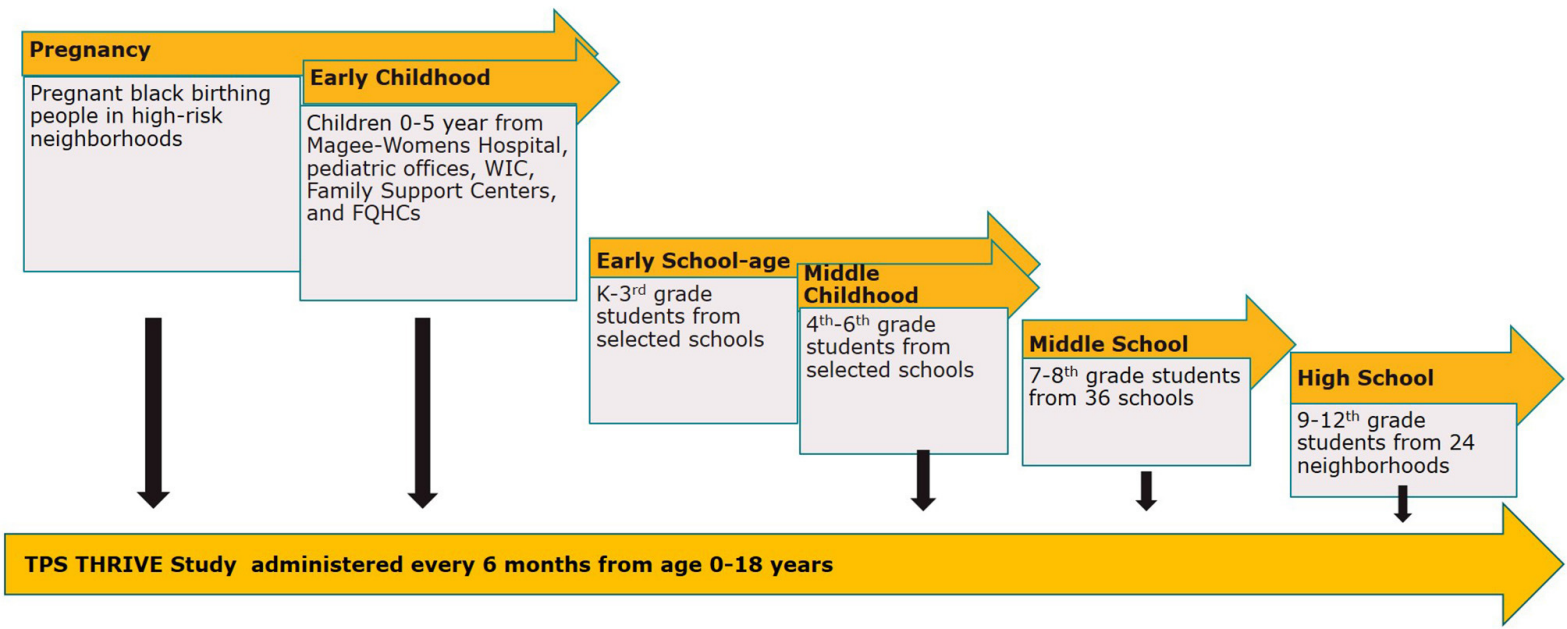
TPS study design. The Pittsburgh Study (TPS) is comprised of the THRIVE longitudinal study and multi-cohort intervention studies. The TPS THRIVE Study will follow children over time from birth through high school, enrolling participants who are part of the intervention studies, as well as siblings and other eligible children in Allegheny County.

Given these persisting inequities, the Pittsburgh Study team brings together groups committed to child health through research and community action. TPS THRIVE is a longitudinal, prospective, observational study that collects cross-cutting child, caregiver, household, and community measures that will follow children over time from birth through high school. THRIVE will enroll participants who are part of the cohort intervention studies as well as siblings and other eligible children in Allegheny County, which is located in Southwestern PA and includes Pittsburgh and its surrounding communities. This approach functions similarly to an accelerated longitudinal cohort design to provide more rapid results and allow earlier translation into practice of effective interventions and guidance for child and adolescent-relevant policies. TPS THRIVE will enroll participants from six cohorts—pregnancy, early childhood, early school-age, middle childhood, and adolescence (middle school and high school), as shown in Figure 2 (TPS Study Design) and described in more detail below.

*Pregnancy*: The Healthy Pregnancy Collaborative (HPC) focuses on reducing racial disparities in prematurity and poor maternal and infant birth outcomes through health system-integrated, technology-based supports for early clinical risk identification and working with community partners to identify and deploy interventions that invest in existing community strengths.

*Early Childhood:* The Early Childhood Collaborative (children 0-5 years) addresses inequities in early child development by providing evidence-based parenting programs to families based on their track record to previously engage low-income families of color in the Pittsburgh community and build on individual family's strengths and resources to allow young children to thrive, setting them on a pathway to flourish as adolescents and adults.

*Early School Age:* The Early School Age Cohort (kindergarten to third grade) has developed an ecosystem approach to improving literacy skills for children in Allegheny County, called “The 3Rs Initiative”: Reading, Racial Equity, and Relationships, supporting high-quality literacy experiences at home, in the community, in classrooms, and in educational leadership.

*Middle Childhood:* The Middle Childhood Cohort (grades 4 through 6), centered on a collaboration between the University of Pittsburgh Center on Race and Social Problems and local, urban public schools to address the school-to-prison pipeline, this study focuses on predominantly low-income students of color to help implement robust relational and restorative climate models (28) to compellingly establish a blueprint for equitable discipline practices in our region.

*Adolescent—Middle School:* The Adolescent—Middle School Cohort (grades 7 and 8) features a middle school study examining the effectiveness of Expect Respect, a teen dating violence and sexual violence prevention program, for middle school age adolescents with histories of exposure to trauma and violence. The two-arm cluster randomized controlled trial in 36 middle schools from local public-school districts and charter school networks will compare violence and bystander behaviors between schools receiving Expect Respect trauma-sensitive support groups compared to schools receiving individual enhanced care assessments.

*Adolescent—High School:* The Adolescent—High School Cohort (ages 13-19) incorporates a community-partnered two-arm cluster randomized trial set in 24 neighborhoods in Allegheny County with concentrated disadvantage to examine the effectiveness of Creating Peace, a trauma-focused, gender-transformative youth violence prevention program that integrates racism and discrimination prevention compared to job readiness training.

### Study Participants

#### Inclusion and Exclusion Criteria

Study eligibility criteria are parents and caregivers who are pregnant or have a child 0-18 years old living with them who reside in Allegheny County, PA. Multiple children per family may enroll in the study between ages 0-18 years old. Children and youth will participate in completing surveys themselves starting at age 8 years old. Currently, the study is open to participants who speak English and Spanish.

#### Recruitment Strategy

Families enrolled in all TPS cohorts are invited to participate in the TPS THRIVE Study. Each of the TPS age cohorts have tailored recruitment strategies based on sample size, intervention site, and intervention delivery. Collaboratives partnered with multiple organizations to recruit pregnant women, new mothers at maternity hospital sites, Special Supplemental Nutrition Program for Women, Infants, and Children (WIC) offices, Federally Qualified Health Centers (FQHCs), and pediatric primary care offices throughout the region. School-based studies recruit families of students enrolled in participating schools and community organizations.

#### Participant Retention

TPS developed partnerships with key leaders in different communities across Allegheny County to serve as TPS Community Ambassadors to enhance connections with communities involved in the study. TPS Community Ambassadors and other community-partner organizations support community outreach and participant recruitment and retention. All participants (children, youth, and caregivers) receive incentives for completing each survey. In addition, TPS offers multiple methods of data collection (in-person, online, by phone) to increase response rates and implement retention best practices for follow-up, reminders, and outreach.

#### Participant Withdrawal

Participants can withdraw from the study for any reason at any time. Changes in child custody, parent/caregiver status, or residence may make the participant ineligible to continue in the study. Study staff will record the reasons for withdrawal and non-retention.

#### Sample Size

Our sampling frame will combine the current TPS study participants across cohorts. For early childhood (0-5 years old), the planned sample size is 7,500. With an estimated response rate of 50% for the first annual THRIVE assessment (3,750 participants), and an 85% retention rate for the second annual assessment, we will have a final sample size of 3,187 children 0-5 years old over 2 years. This will provide the ability to detect a 0.08 difference in parent thriving Brief Inventory of Thriving (BIT) score and 5.5% difference in prevalence of child flourishing with a power of 85% (alpha = 0.05).

For school-age children and adolescents, the planned sample size is 4,930. With an estimated response rate of 50% for the first annual assessment (2,465 participants), and an 85% retention rate for the second annual assessment, we will have a final sample size of 2,095 children and youth 6-18 years old over 2 years. For our sample of 6- to 18-year-old children, our study will have the power to detect a 1-point difference in our main outcome of youth thriving (85% power, alpha of 0.05; BIT mean score (SD): 3.71 (0.78)).

### Study Comparison Groups

TPS combines family, school, and neighborhood-level interventions, targeting contexts most relevant to improving child outcomes by developmental age. For baseline survey data, descriptive analyses will assess mean levels of outcomes across subgroups (by age, sex, race/ethnicity, and family income). We will examine changes over time using paired *t*-tests and adjusted linear regression models for continuous outcomes and McNemar tests and adjusted logistic regression models for categorical variables.

### Study Outcomes and Measures

Table 2 provides an overview of the outcomes and assessments mapped to the conceptual framework domains of thriving.

**Table 2:** Cross-cutting study measures, assessments, and ages.

**Domain**	**Measure/data source**	**Respondent**	**Ages**
**Strong bodies**			
Overall health	PROMIS global health (29)	Caregiver, youth	0–18
Physical activity	PROMIS physical activity (30) Outdoor time	Caregiver, youth	0–18
Eating/nutrition	YRBS (31)	Caregiver, youth	0–18
Height and weight	Self-report, HER	Caregiver, youth	0–18
Sleep	PROMIS sleep health—disturbance and impairment (32)	Caregiver, youth	0-18
Screen time	NSCH screen time (33)	Caregiver, youth	0–18
Health behaviors	YRBS (31)	Youth	12–18
Alcohol, tobacco, and drug use	YRBS (31)	Youth	8–18
Sexual activity	YRBS (31)	Youth	12–18
**Strong minds**			
Child mental health	NSCH mental health diagnoses (33) PROMIS anxiety and depression (34)	Caregiver, youth	0–18
School readiness	NSCH healthy and ready to learn (33)	Caregiver	3–5
School connectedness	YRBS (31)	Caregiver, youth	6–18
Parent engagement	YRBS (31)	Caregiver	6–18
School attendance	YRBS (31)	Caregiver, youth	6–18
Academic performance	YRBS (31), school records	Caregiver, youth	6–18
**Positive identity and self-worth**			
Learning behaviors	PROMIS curiosity, persistence, and adaptation	Caregiver	0–5
Thriving, future orientation/hope	NSCH Flourishing Index (33) Brief Inventory of Thriving (35)	Caregiver, youth	6–18
Meaning and purpose	PROMIS meaning and purpose (36)	Caregiver, youth	6–18
Resilience	Youth Thriving Survey resilience subscale (37)	Caregiver, youth	6–18
**Fun and happiness**			
Child positive affect	PROMIS positive affect (38)	Caregiver, youth	0–18
**Caring family and relationships**			
Family relationships	PROMIS family relationships (39)	Caregiver, youth	0–18
Family routines	CDC National Survey of Early Childhood - Family routines (40)	Caregiver, youth	0–18
Family resilience	NSCH family resilience (33)	Caregiver, youth	0–18
Peer relationships	PROMIS peer relationships (41)	Caregiver, youth	0–18
Caregiver social support	PROMIS instrumental, emotional, and informational support (42)	Caregiver	0–18
Caregiver stress	Parental perceived stress (43)	Caregiver	0–18
Caregiver thriving	Human Flourishing Scale (44)	Caregiver	0–18
Caregiver relationships	Dyadic Adjustment Scale (45)	Caregiver	0–18
**Safety**			
Neighborhood safety	Perceived Neighborhood Safety Scale (46)	Caregiver, youth	0–18
Exposure to violence and bullying	YRBS (31)	Caregiver, youth	8–18
**Vibrant communities**			
Neighborhood satisfaction	Neighborhood Satisfaction Scale (47)	Caregiver, youth	0–18
Collective efficacy	Collective efficacy (48)		
Access to green space	PHENOTYPE items (49)	Caregiver, youth	0–18
Transportation	Protocol for Responding to and Addressing Patient's Assets, Risks, and Experiences (PRAPARE) (50)	Caregiver	0–18
Safety	PRAPARE relationships (50)		
**Healthy environments**			
Housing quality	American Housing Survey (47)	Caregiver	0–18
Water safety	American Housing Survey (47)	Caregiver	0–18
Lead exposure	Blood lead levels	Caregiver, EHR	0–18
Air quality Exposure to smoke	Secondary data NSCH Exposure to Smoke (33)	Caregiver	0–18
Food security	USDA Food Security Screener (51)	Caregiver	0–18
**Racial justice, equity, inclusion**			
Racism and discrimination	Multigroup Ethnic Identity Measure (52), Perceived Discrimination Scale (53)	Caregiver, youth	0–18
Socialization	Cultural Socialization Behaviors Measure (54)	Caregiver	0–18
Religion/spirituality	Spiritual well-being (55)	Caregiver, youth	0–18
**Healthcare services**			
Usual source of care and service use	NSCH usual source of care and health service use (33)	Caregiver	0–18
Health insurance and costs	NSCH health insurance (33)	Caregiver	0–18
Trust in medical providers	Abbreviated Trust in Physicians Scale (56)	Caregiver	0–18
Immunizations	Parent Attitudes about Childhood Vaccines Survey (57)	Caregiver	0–18
Primary Care Communication	Parents perceptions of pediatric primary care quality (58), Interpersonal Processes of Care Survey (59)	Caregiver report	0–18
Health information	Consumer Health Information (60)	Caregiver report	0–18
Health literacy	Abbreviated Health Literacy Measure (61)	Caregiver report	0–18

*Family demographic data will also be collected including child and caregiver age, race/ethnicity, primary language, and gender identity; primary and secondary caregiver income, employment, education, use of other social services; residential history; Household size, number of children*.

#### Primary Outcome Measures

Primary outcomes of TPS THRIVE are child/youth thriving measures across developmental stages. The early childhood (ages 0-5 years old) primary outcome is the National Survey of Children's Health (NSCH) (3) measure of thriving/flourishing. For school-age children and youth (aged 6-18 years old), the primary outcome is thriving score on the Brief Inventory of Thriving (BIT), a 10-item assessment of youth psychological well-being (35).

#### Secondary Outcome Measures

Secondary outcome measures for early childhood (0-5 years) are the National Institutes of Health (NIH) Patient Reported Outcome Measure Information System (PROMIS) (2) measures of thriving/flourishing, including curiosity, persistence, adaptability, supportive relationships, and positive affect, as well as the NSCH Healthy and Ready to Learn measure of school readiness (62). For school-age and adolescent children, secondary outcomes are future orientation, PROMIS measures of meaning and purpose (36), and academic proficiency (reading and math scores) and achievement (grade completion and graduation).

#### Social Influences on Health Measures

Intermediate outcomes include social influences on health including cross-cutting study measures and assessments of family, school, and community factors that influence child and youth thriving outcomes including:

- Influence of policy and place on child thriving, such as neighborhood-level collective efficacy (48), experiences of racism/discrimination (52, 53), access to community resources, and neighborhood safety (46).
- Influence of physical and social environments on child thriving, including water/air/housing quality, child/school experiences, and positive and adverse child experiences (47).
- Influence of health services delivery on child thriving, including access to health care, trust in health care providers (56), and health literacy (61) and communication (59).

### Study Procedures and Data Collection

#### Primary Data Collection

The TPS THRIVE study is administered online, with options for phone and in-person completion for participants who may not have access to technology or have other barriers to online completion. Planned future assessments include physical assessments, biomarkers, genetic information, and environmental samples. Study questionnaires and data collection forms can be found on the Study SharePoint site.

#### Secondary Data Collection

The study also combines parent/caregiver and youth (ages 8-18 years) reported survey data with administrative and secondary data, including child and caregiver electronic health records (EHRs), birth certificates, and geospatial community data. Enrolled participants are asked for permission to access health service encounters and assessments from medical records, health and social service use from health and human service departments, school record data, and other administrative secondary data. This Healthy Environment, Strong Bodies Committee synthesizes local data sources, research studies, and collaborative initiatives focused on selected exposures of high interest to the community: psychosocial stress, air quality, water quality, chemical contaminants, infectious disease, green space, and food security.

#### Training and Certification

Study personnel will be trained within each intervention study and centrally in the study protocol requirements, including standardized measurement and eliciting information from study participants in a uniform reproducible manner. The TPS Study Coordinator and Cohort Coordinators will review how to collect data and conduct the procedures at each visit in detail. All study staff including community members will complete required training in human subjects research ethics, data privacy, and data security procedures.

### Data Management

#### Data Entry and Tracking

The Center for Research on Health Care Data Center (CRHC-DC) serves as the Data Core for TPS. The Data Core develops strategies for study data entry and management. Data are entered electronically via a password-protected web-based data entry system, but paper versions of forms are provided for manual entry in case of technical problems. Our web-based system, developed using ASP.NET programming, adheres to guidelines set by FDA 21 CFR Part 11, which mandates practices with respect to data access, data confidentiality, password protection, audit trails, validation, and direct data entry. The data entry process begins during the online participant registration into TPS which generates a participant ID at initiation to track participants, link data, and maintain data confidentiality. The tracking system produces a schedule of data collection for each participant. TPS study coordinators log into the data entry and tracking system, enter the participant's study ID, and choose the visit to be viewed. The appropriate set of forms are generated for that participant based on the visit number. To minimize missing data, all study forms have certain key fields that are required before forms can be submitted. The web-based system can link each TPS study participant across multiple cohorts and across multiple family members.

#### Data Monitoring

The TPS web-based data management system will track the progress of participants throughout individual studies. The tracking and reporting systems are integrated within the web-based data management system. They include programmed follow-up intervals to allow TPS study personnel to determine which participants are due for a particular visit. The customized tracking system allows TPS to generate reports for monitoring of study progress on recruitment, visit completion, retention, and safety. The TPS Equity, Ethics, and Community Accountability committee will review reports of any adverse events and potential harms to study participants and participating communities and populations.

#### Data Protection and Access

Personal information about potential and enrolled participants are collected and maintained to protect confidentiality before, during, and after the study. Online data access is restricted to authorized study personnel through the password protected data system. Any hard-copy study-related information will be stored securely at the study site, and all participant information will be stored in locked file cabinets in areas with limited access. All reports, data collection, process, and administrative forms will be deidentified using a coded identifier to maintain participant confidentiality. All records that contain names or other personal identifiers, such as locator forms and informed consent forms, will be stored separately from study records identified by code number. Contact forms and any other listings that link participant ID numbers to other identifying information will be stored in a separate folder with limited access. The data team will have access to the final study dataset. Limited data sharing agreements have been developed to link participant data with secondary data with participant permission.

#### Data Quality

During data entry, several strategies are employed to ensure quality of data: use of standard methods of data collection and recording, careful programming of the data management system, detailed documentation of computer operations and data editing procedures, and regular meetings with project staff to review any changes in procedure. TPS Data Core verifies all data, programs out-of-range data checks into data entry fields, and evaluates the full data process within and across forms. TPS Data Core personnel check outlying values from enrollment of the first participant to the data-cleaning phase, conducting logical checks and analyzing outliers. Audit logs will track changes to information previously submitted and recorded on electronic forms to ensure data integrity. Information about the person responsible for the change, the date of the change, the previous entry in the data field, the new entry in the data field, and the reason for the change are recorded and displayed in the electronic forms audit trail.

### Data Analysis

The Biomedical Statistics and Data Science Lab also collaborates with the CRHC-DC to conceptualize and implement statistical analysis plans and facilitates interpretation of results. The Strengthening the Reporting of Observational Studies in Epidemiology (STROBE) statement (63) for observational studies and the Methodology Standards developed by the Patient-Centered Outcomes Research Institute (64) (including all cross-cutting standards) were employed in the design of the study.

For each research question, the team will develop a causal diagram (65) for the exposure-outcome relationships of interest, including a list of the predictors, confounders, mediators (i.e., measures on the causal pathway between the exposure and outcome), and potential effect modifiers of the exposure-outcome relationship. The causal diagram will be used to specify standard regression models, including linear regression for continuous outcomes, logistic regression for binary outcomes, Poisson or negative binomial regression (depending on the skewness of the outcome distribution) for counts and rates, and mixed models with a random intercept (in combination with the previously-specified types of regression models) to account for within-subject correlation (66). The included variables and their parameterization in the model (e.g., interactions or non-linear terms) are guided by clinical knowledge of the hypothesized relationship from the causal graphs. In cases where the clinical importance of a variable or subset of variables is unclear to investigators, significance tests (e.g., partial *F*-tests or likelihood ratio tests) will be used to assess significance against the reduced model (i.e., terms deemed a-priori as clinically important). In some cases, the number of variables and complexity of the model may be limited by the available sample size.

#### Subgroup Analyses

Given the focus of TPS on building health equity in child outcomes, we will conduct subgroup analyses by child race/ethnicity, child gender identity, and family income level to determine any differential access to programs. Both unadjusted and adjusted analyses will be performed. Adjusted analyses will include key demographic variables and other variables identified as potential confounders of the relationship between the exposure and outcome measures.

#### Missing Data and Potential Bias in Attrition

Given the pragmatic nature of these studies, missing data and attrition can be expected. The degree of missingness will be described for all variables, and summaries of the other variables will be used to describe differences between observations with missing and non-missing values for key variables to assess the likelihood of missing at random. For variables that are deemed to be missing at random, imputation or multiple imputation may be used (depending on the percentage of missingness) to improve precision. Lost to follow-up will be described consistent with STROBE guidelines.

## Discussion

With a multi-sector, transdisciplinary team of collaborators, TPS THRIVE is a community-partnered multi-cohort study designed to provide longitudinal data about interrelated dimensions of child thriving in the context of family, school-based, and community programs. In addition, the study is developing an infrastructure to build community capabilities, including training in leadership, data literacy, and antiracism to enhance research skills and ongoing professional development among community members.

### Strengths and Limitations

Strengths of TPS THRIVE include its grounding in a comprehensive, community-informed conceptualization of childhood thriving and its asset-based approaches in building on the strengths of families and neighborhoods in supporting childhood thriving. This transdisciplinary collaboration integrates experts in pediatrics, developmental science, education, health services research, community health, public health, social work, biostatistics, epidemiology, behavioral health, engineering, and biological and environmental science along with community organizations in an innovative community-partnered research model. By integrating community organizations and members, this study builds on positive practices already present in our community, identifying interventions that have community relevance and contextual validity.

Limitations of this study include self-report bias of survey data, selection bias, and lack of randomized, control groups for some of the cohort intervention studies. Secondary data and direct assessments will be used when available to supplement self-reported survey data. Propensity score matching to account for group differences will be used when randomized comparison groups are unavailable. Different sample inclusion/exclusion criteria may make it difficult to compare children across cohorts over time.

### Implications

TPS has the potential to increase our understanding of child thriving and deepen community partnerships and engagement while also yielding interventions likely to be sustainable and workable for local communities. Incorporating the importance of history, culture, community, and multiple dimensions of child health, TPS seeks to understand complexities at the root of child health and educational disparities. Documented race, gender, and economic inequities (1, 67) in our region will be defined using ecological data, developmental systems, and equity frameworks. Results from TPS will be used to develop and adapt interventions based on the needs of participants and effectiveness at improving thriving outcomes and reducing disparities. New interventions will be identified and selected based on community input and data analyses after current interventions have been evaluated and revised or integrated into community, clinical, and academic settings. In this way, TPS will provide critical insights to guide future design and pilot testing of family and community interventions to foster child thriving and eventually change policy governing child well-being.

## Ethics and Dissemination

### Ethical Review and Approval

TPS THRIVE has been reviewed and approved by the University of Pittsburgh Institutional Review Board (IRB): STUDY19120093. Written informed consent was obtained from all adult participants who have already enrolled in the study and will be obtained from all future adult participants. Children and youth will provide verbal assent to participate in the study. Any study protocol modifications will be submitted to the IRB prior to being implemented. All study investigators, research personnel and study participants (if appropriate) will be informed of study changes and all updated materials are posted in the TPS Regulatory Binder which is accessed online via SharePoint.

Since this research study involves no greater than minimal risk as assessed by the University of Pittsburgh IRB, a formal Data Monitoring Committee is not required. However, the TPS Equity, Ethics, and Community Accountability committee focuses on review of all study procedures, monitoring study progress to ensure adherence to TPS shared principles. In addition, the following auditing activities are conducted by the investigators on a weekly basis: (a) systematic review of procedures to ensure that all study activities are conducted appropriately and protocols followed; (b) ensure that any participants disclosing abuse, violence, or suicidal intent/self-harm receive appropriate referrals to services and that mandated child abuse reports are made when appropriate; and (c) monitor staff performance related to protection of privacy, confidentiality, maintenance of secure databases, and study procedures designed to reduce the risk of potential breaches of confidentiality. Any adverse events or protocol deviations associated with the study will be reported to the University of Pittsburgh IRB.

Study recruitment and data collection procedures have been modified due to COVID-19 restrictions on in-person visits to occur virtually when possible. The study has implemented research safety protocols and plans to protect research teams and participants as well as continuous data safety monitoring.

#### Community Research Training and Reciprocity

All TPS research team members, including community partners, are required to complete training on the ethical and regulatory aspects of human subjects' research. Scientific committee members from the community receive training in principles of conduct of ethical research using the Community Partner Research Ethics Training (CPRET) program. Developed by the University of Pittsburgh Clinical and Translational Science Institute (CTSI) (68), University of Pittsburgh Human Research Protection Office and Community Research Advisory Board, this unique program allows research investigators to tailor research ethics training for their specific research study and the role that their community partners will play in the research. CPRET allows research investigators to discuss scenarios that raise ethical concerns relevant to their research study.

### Data Dissemination and Reporting

The TPS THRIVE Study dissemination strategy was developed in partnership with community members, and all study presentations and publications will include 50% community co-authors. Study results will be communicated to study participants, community members, healthcare professionals, the public, and other TPS stakeholders through publications, monthly meetings and newsletters, and biennial retreats. Deidentified, aggregate level data will be accessible via the TPS Child Health Data Hub. A process for reviewing requests for study datasets has been established and will include review by a panel of study leads and community partners.

## Ethics Statement

The studies involving human participants were reviewed and approved by University of Pittsburgh Institutional Review Board. Written informed consent to participate in this study was provided by the participants' legal guardian/next of kin.

Pittsburgh Study Committee Co-leads (alphabetical): Rev. Paul Abernathy; Debra Bogen, MD; Janet Catov, PhD, MS; Emilie Delestienne, MPH; Barbara Fuhrman, PhD; Richard Garland, MSW; Robert Gradeck, MA; Catherine L. Haggerty, PhD, MPH; James Huguley, PhD; Tamar Krishnamurti, PhD; Amy Malen, MPP; Vanessa Mayers-Snyder; Shallegra Moye, MPH; Michelle Naccarti-Chapkis; Mary Ohmer, PhD; Tomar Pierson-Brown, JD, LLM; Kristin N. Ray, MD, MS; Daniel Shaw, PhD; Jada Shirriel, MS; Shannah Tharp-Gilliam, PhD; Shannon Wanless, PhD; Dannai Wilson, MS; Todd Wolynn, MD.

## Author Contributions

AE contributed to the design of the study and served as lead author on the manuscript. DL developed analytic plan for data interpretation and was a major contributor in writing the manuscript. KA developed plan for data acquisition and analysis and was a major contributor in writing the manuscript. JB made substantial contributions to the conception and design of the study and study's community engagement framework. VC made substantial contributions to the conception and design of the study and study's community engagement framework. JN developed IRB protocols, study materials, and helped draft the manuscript. FS made substantial contributions to study's community engagement framework and reviewed the final manuscript. TD made substantial contributions to the overall conception and design of the study and reviewed the final manuscript. EM made substantial contributions to the overall conception and design of the study. TPS Scientific Committee Co-Leads contributed to overall design of study and wrote sections of the manuscript. All authors have read and approved the final manuscript.

## Funding

This work was supported in part by the Grable Foundation; the Shear Family Foundation; UPMC Children's Hospital of Pittsburgh; and the Children's Hospital of Pittsburgh Foundation. The study sponsor (University of Pittsburgh) and funders did not have any role in study design; collection, management, analysis, and interpretation of data; writing of the report; and the decision to submit the report for publication, including whether they will have ultimate authority over any of these activities.

## Conflict of Interest

FS is employed by YogaRoots On Location, LLC. The remaining authors declare that the research was conducted in the absence of any commercial or financial relationships that could be construed as a potential conflict of interest.

## Publisher's Note

All claims expressed in this article are solely those of the authors and do not necessarily represent those of their affiliated organizations, or those of the publisher, the editors and the reviewers. Any product that may be evaluated in this article, or claim that may be made by its manufacturer, is not guaranteed or endorsed by the publisher.

## Footnotes

^1^We have capitalized Black, White and other racial/ethnic group names as required by the journal formatting requirements. However, our study standard is to capitalize Black and not white to recognize the shared history and identity of Black people. This approach, recommended by other academic publishers, also highlights that white does not represent a shared history and culture in the way Black does, and resists the capitalization long used by hate groups.

## Abbreviations

CDC: Centers for Disease Control and Prevention
CFR: Code of Federal Regulations
CPPR: Community-partnered Participatory Research
CRHC: University of Pittsburgh Center for Research on Health Care
CTSI: University of Pittsburgh Clinical and Translational Science Institute
EHR: Electronic Health Record
WIC, Special Supplemental Nutrition Program for Women, Infants: and Children
FQHC: Federally Qualified Health Center
FDA: U.S. Food and Drug Administration
NIH: National Institutes of Health
NSCH: National Survey of Children's Health
PROMIS: Patient Reported Outcome Measure Information System
STROBE: Strengthening the Reporting of Observational Studies in Epidemiology
TPS: The Pittsburgh Study
UPMC: University of Pittsburgh Medical Center
YRBS: Youth Risk Behavior Survey.
